# Potential Role of Yeast Strains Isolated from Grapes in the Production of Taurasi DOCG

**DOI:** 10.3389/fmicb.2016.00809

**Published:** 2016-05-27

**Authors:** Maria Aponte, Giuseppe Blaiotta

**Affiliations:** ^1^Sezione di “Microbiologia”, Dipartimento di Agraria, Università degli Studi di Napoli Federico IINapoli, Italy; ^2^Sezione di “Scienze della Vigna e del Vino”, Dipartimento di Agraria, Università degli Studi di Napoli Federico IIAvellino, Italy

**Keywords:** grapes, yeast microflora, Aglianico, identification, biotyping, wine fermentation

## Abstract

Twelve samples of Aglianico grapes, collected in different locations of the Taurasi DOCG (Appellation of Controlled and Guaranteed Origin) production area were naturally fermented in sterile containers at room temperature. A total of 70 yeast cultures were isolated from countable WL agar plates: 52 in the middle of the fermentation and 18 at the end. On the basis of ITS-RFLP analysis and ITS sequencing, all cultures collected at the end of fermentations were identified as *Saccharomyces (S.) cerevisiae*; while, the 52 isolates, collected after 1 week, could be referred to the following species: *Metschnikowia* (*M*.) *pulcherrima*; *Starmerella* (*Star*.) *bacillaris*; *Pichia* (*P*.) *kudriavzevii*; *Lachancea* (*L*.) *thermotolerans*; *Hanseniaspora (H.) uvarum*; *Pseudozyma (Pseud.) aphidis*; *S. cerevisiae*. By means of Interdelta analysis, 18 different biotypes of *S. cerevisiae* were retrieved. All strains were characterized for ethanol production, SO_2_ resistance, H_2_S development, β-glucosidasic, esterasic and antagonistic activities. Fermentation abilities of selected strains were evaluated in micro-fermentations on Aglianico must. Within non-*Saccharomyces* species, some cultures showed features of technological interest. Antagonistic activity was expressed by some strains of *M. pulcherrima, L. thermotolerans, P. kudriavzevii*, and *S. cerevisiae*. Strains of *M. pulcherrima* showed the highest β-glucosidase activity and proved to be able to produce high concentrations of succinic acid. *L. thermotolerans* produced both succinic and lactic acids. The lowest amount of acetic acid was produced by *M. pulcherrima* and *L. thermotolerans;* while the highest content was recorded for *H. uvarum*. The strain of *Star. bacillaris* produced the highest amount of glycerol and was able to metabolize all fructose and malic acid. Strains of *M. pulcherrima* and *H. uvarum* showed a low fermentation power (about 4%), while, *L. thermotolerans, Star. Bacillaris*, and *P. kudriavzevii* of about 10%. Significant differences were even detected for *S. cerevisiae* biotypes with respect to H_2_S production, antagonistic activity and β-glucosidase activity as well as for the production of acetic acid, glycerol and ethanol in micro-vinification experiments.

## Introduction

Wine composition and quality are affected by several intrinsic and extrinsic variables, many of which are microbiologically mediated. Spontaneous alcoholic fermentation of grape must is a complex process owing to metabolic activities of different groups of microorganisms including filamentous fungi (i.e., *Botrytis* spp.), yeasts, and bacteria (lactic and acetic acid bacteria) originating from grapes, soil, and cellar equipment (Mills et al., 2008). The physiological properties of these complex microbial consortia lead to the formation of metabolites and to the transformation of grape molecules, thus influencing the sensorial properties (color, aroma, flavor, structure, and body) of the final product (Pretorius, 2000; Fleet, 2003). Due to the sequential action of different yeast species/strains, naturally present on the berries grapes or in the winery, the outcome of spontaneous alcoholic fermentation is difficult to predict and therefore, results are often unreproducible (Pretorius, 2000). To address this issue, many winemakers use pure yeast cultures (starters) of *S. cerevisiae* or *S. bayanus* species, which are inoculated into the must after pressing. The use of starter cultures allows a more rapid and complete grape must fermentation and a higher degree of reproducibility in the atmosphere of specific wines can be achieved (Pretorius, 2000; Fleet, 2008; Suarez-Lepe and Morata, 2012). However, there is some controversy about the use of commercial wine yeasts due to the lack of some desirable traits provided by natural or spontaneous fermentation (Pretorius, 2000). Moreover, the continuous use of a limited number of strains as commercial starter cultures by wine industry is causing the erosion of the microbial diversity. The study and the preservation of the wine yeasts biodiversity have recently become matter of growing interest (Di Maio et al., 2012). The maintenance of the biological patrimony is essential to obtain starter strains able to fully develop the typical sensory profile of wines originating from different grapevine cultivars, as well as to preserve a gene pool of paramount importance for any yeast-mediated process (Pretorius, 2000; Marinangeli et al., 2004). Such criticism is providing new challenges to enhance the appeal and value of wine produced by this fermentation technology. As reviewed by Fleet (2008), this may be achieved by selecting novel yeast starter cultures from natural wine environment and by leading the fermentations with mixtures of yeast species (including *Saccharomyces* and non-*Saccharomyces*) and strains, for flavor modulation, volatile acidity decreasing, malic and lactic acids production or degradation.

The present survey was focused on Taurasi DOCG (Appellation of Controlled and Guaranteed Origin), a wine produced within a small area of the Campania Region (Irpinia district) by a starter-led fermentation technology. Taurasi DOCG, as reported in the production specifications (Ministerial Decree 11 March 1993; G.U. n. 72 of 27 March 1993), is a red wine manufactured by *Vitis vinifera* cv. *Aglianico* (at least 85%) exclusively cultured in 17 municipalities (Taurasi, Bonito, Castelfranci, Castelvetere sul Calore, Fontanarosa, Lapio, Luogosano, Mirabella Eclano, Montefalcione, Montemarano, Montemileto, Paternopoli, Pietradefusi, Sant'Angelo all'Esca, San Mango sul Calore, Torre le Nocelle e Venticano) of the Avellino province. To explore the natural yeast diversity, grapes from 12 different vineyards were analyzed. Molecular methods were applied for isolates identification as well as for strains biotypization within the *S. cerevisiae* species. The potential winemaking role of isolated yeast strains was assessed by evaluation of oenological traits and of the behavior in micro-fermentation trials in Aglianico must.

## Materials and methods

### Sampling and yeast isolation

Samples of Aglianico grapes were collected (end of October 2012) in 12 vineyards located in the municipalities where this variety is cultivated (Table 1). The origin and °Babo of grape samples are reported in Table 1. Samples (about 20 bunches) were collected by using sterile gloves along the two diagonals of the vineyard, placed in sterile plastic bags and transferred in the laboratory within few hours. Grapes were manually pressed in the collection bag, and, after the addition of potassium metabisulfite (100 mg/kg), were incubated at room temperature (18–22°C). During incubation the sugar content (°Babo) was monitored and, after 9 days of fermentation, must samples were analyzed. After sampling, partially fermented musts were combined into one sterile container and left to ferment until complete sugars consumption (mix-wine).

**Table 1:** **Origin of grape samples and basic physico-chemical characteristics of the musts**.

**Grape Sample**	**Origin (Municipality)**		**Must characteristics**		
			**°Babo**	**pH**	**Total acidity^a^**
1	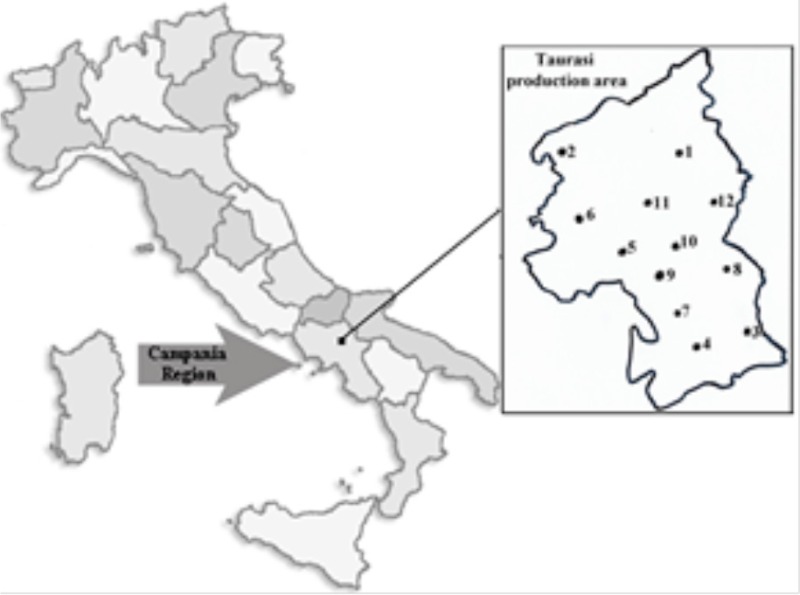	Mirabella Eclano	20.9	3.18	9.57
2		Pietradefusi	20.8	3.41	9.26
3		Castelfranci	19.4	3.26	9.65
4		Montemarano	20.7	3.18	9.58
5		Lapio	20.3	3.22	9.05
6		Montemileto	21.6	3.21	8.78
7		Castelvetere sul Calore	20.6	3.35	8.56
8		Paternopoli	21.6	3,32	9.36
9		San Mango sul Calore	19.8	3.08	11.21
10		Luogosano	19.0	3.11	10.20
11		Taurasi	20.8	3.34	8.79
12		Fontanarosa	19.8	3.21	10.26

*Location of Taurasi production area and of vineyards where grape sampling was carried out is reported on the map (collection sites are indicated by numbers)*.

a *g/l of tartaric acid (25 ml of wine sample and 0.25 N NaOH)*.

Must samples and mix-wine were serially diluted in quarter strength Ringer's solution (Oxoid, Basingstoke, UK) and spread-plated on WL-nutrient agar (Oxoid). After incubation at 28°C for 5 days, countable plates (15–150 colonies/plate) were used for viable counts and yeasts isolation. Colonies showing different morphology and/or color were all selected, independently by their number. In mix-wine sample, all colonies (n°18) grown in one of the two countable plates were considered. Cultures were purified by repetitive streaking on WL-nutrient agar.

Yeast cultures were preserved on WL-nutrient agar slants, stored at 4°C and sub-cultured every 3 months. Before each test, strains were cultured twice in YPD (yeast extract 10 g/l, peptone 20 g/l, dextrose 20 g/l).

### Yeast strains molecular identification and typing

DNA was isolated as previously reported by Aponte and Blaiotta (2016). Preliminary molecular identification of yeast strains was achieved by ITS (ITS1-5.8S-ITS2)-rDNA RFLP (Esteve-Zarzoso et al., 1999; Csoma and Sipiczki, 2008) analysis using restriction endonucleases *Hae* III, *Hinf* I, and *Cfo* I. In addition, enzymes *Dde* I and *Mbo* I were used for the characterization of *Hanseniaspora* and *Candida* spp, respectively. The identification of non-*Saccharomyces* cultures was obtained by ITS-rDNA sequencing. Genetic diversity within *Saccharomyces* isolates was assessed by Interdelta analysis (Legras and Karst, 2003).

### Yeast strains technological characterization

Ethanol tolerance was evaluated in YPD broth containing ethanol concentrations ranging from 4 to 15% (v/v). After incubation at 20°C for 72 h, growth was assessed by spectrophotometry at white light (600 nm). Sulfur dioxide (SO_2_) tolerance was evaluated in YPD broth adjusted at pH 3.30 with tartaric and malic acids (1:1) and containing potassium metabisulfite concentrations ranging from 50 to 200 mg/l. Growth was evaluated, after incubation at 20°C for 72 h, by spectrophotometry at white light (600 nm). Hydrogen sulfide (H_2_S) production was estimated on Biggy agar (Oxoid) after incubation at 28°C for 48 h. For browning description, the following codes were used: low production, snow-white color; medium production, hazelnut-brown color; high production, rust-coffee color (Aponte and Blaiotta, 2016). Type of growth was estimated in tyndallized (100°C × 5 min × 3 times) must (21°Brix, pH 3.50) after 4 days at 25°C. Antagonistic activity was assessed as described by Sangorrin et al. (2001) using *S. cerevisiae* CECT 1890 as sensitive strain.

β-glucosidase activities were evaluated on media containing cellobiose (CELL), 4-methylumbelliferyl-b-D-glucopyranoside (MUG), arbutin (ARB), esculin (ESC), or p-nitrophenyl β-D-glucopyranoside (pNPG) (Fluka, Milan, Italy), according to the method proposed by Fia et al. (2005) and Hernandez et al. (2002). Esterase activity was evaluated on a medium containing Tween 80 as described by Slifkin (2000).

In order to estimate the percentage of similarity among isolates, data were subject to cluster analysis (Average Linkage Method). A correlation matrix was constructed using the formula described by Upholt (1977) and Nei and Li (1979): F_xy_ = (2n_xy_)/(n_x_+n_y_) where F_xy_ is the proportion of common molecular markers of molecular biotypes compared (x and y), n_xy_ is the number characters shared by both isolates x and y and n_x_ and n_y_ are the total of number characters of observed in isolates x and y, respectively [in our case (n_x_ + n_y_) = (10+10) = 20]. The resulting correlation matrix was analyzed by Systat 5.2.1 software.

### Fermentation performances of selected yeast strains

Fermentation vigor (FV) and fermentation power (FP) were evaluated in micro-fermentation trials in Aglianico must (°Brix 24, pH 3.09; total acidity 9.98 g/l of tartaric acid). Strains, cultured twice in YPD medium, were used to inoculate (about 6 Log CFU/ml) 100 ml of tyndallized (100°C for 3 min for 3 times) must in 250 ml Erlenmeyer flasks closed with a Müller valve filled with sulfuric acid. During incubation (3 days at 23°C), flasks were handle stirred for 30 s every 12 h. Weight loss, due to CO_2_ escaping from the system, was quantified to monitor the fermentation kinetics. Fermentation was considered concluded when no weight loss was any longer recorded within 24 h. FV was expressed as grams of CO_2_ produced in 100 ml of must during the first 72 h of fermentation, while FP was expressed as grams of CO_2_ produced until the end of fermentation. Each trial was performed in triplicate. At the end of micro-fermentations, concentrations of citric, tartaric, malic, lactic, and succinic acids and of glucose, fructose, glycerol, 2,3-butanediol and ethanol were determined by HPLC analyses as previously described by Aponte and Blaiotta (2016).

## Results

The aims of the present study were the yeast microbiota exploration of Aglianico grapes, grown in the Taurasi DOCG area and the evaluation of potential technological contribute of autochthonous yeast strains in winemaking. Grapes were sampled in 12 different vineyards located in area of production of this typical wine (Table 1); physico-chemical characteristics of relative musts are reported in Table 1. Musts showed a high sugar content (20.4 ± 0.8 °Babo as average value) and were characterized by low pH (3.2 ± 0.1) and high total acidity (9.5 ± 0.8 g/l). After 9 days of fermentation at room temperature, musts showed highly different residual sugar contents (°Babo from 7 to 13) and alcoholic degrees (Malligand ebullioscope degree from 2 to 8% vol/vol) (Table 2). In fact, 9 musts out of 12 still contained more than a half of the initial sugar content. Viable yeast counts ranged from 6.4 to 8.2 Log CFU/ml (Table 2).

**Table 2:** **Physico-chemical and microbiological characteristics of musts and mix wine after partial fermentation (9 and 30 days, respectively)**.

**Must sample**	**°Babo**	**Ethanol (% vol/vol)^a^**	**Yeast loads (Log CFU/ml)^b^**	**No. of isolates^c^**	**Species**^**d**^						
					***M. pulcherrima***	***Star. bacillaris***	***P. kudriavzevii***	***L. thermotolerans***	***H. uvarum***	***Pseud. aphidis***	***S. cerevisiae***
1	7.8	7.9	7.21 ± 0.21	4							4
2	13.8	4.2	8.16 ± 0.01	7				1		2	4
3	14.3	3.1	7.36 ± 0.05	4		1		1			2
4	17.3	2.1	7.33 ± 0.00	5	1				2		2
5	13.8	3.9	7.25 ± 0.15	5			3	1	1		
6	17.3	2.6	6.37 ± 0.02	2	2						
7	15.1	3.3	6.81 ± 0.51	5	1			1	1		2
8	10.8	6.4	6.80 ± 0.52	5					1		5
9	14.7	3.1	6.90 ± 0.44	3	1			2			
10	6.9	7.3	7.80 ± 0.06	4							4
11	11.7	5.4	7.77 ± 0.19	4							4
12	6.9	7.8	8.05 ± 0.04	4							4
Mix-wine	1.8	12.8	5.30 ± 0.06	18							18

a *Ebullioscopic (Malligand)*.

b *Counts on WL Nutrient agar (28°C for 5 days)*.

c *Selection on the basis of colony colur and morphology from countable plates (15-150 colonies/plate)*.

d *Identifications obtained by ITS-RFLP and ITS sequencing analyses (see Supplementary Table S1)*.

The mix-wine was obtained by joining partially fermented musts whose fermentation was allowed to proceed for further 30 days, namely until the sugar content did not change for 48 h. The mix-wine reached an alcoholic degree of 12.8% vol/vol (Table 2) and still contained a high concentration of residual sugars (10.7 g/l of glucose and 11.2 g/l of fructose) and acetic acid (3.2 g/L) as determined by HPLC analysis. Yeast loads were still high as well (around 5 Log CFU/ml). Fifty-two yeast cultures were isolated from partially fermented musts, on the basis of colony morphology and color on counting plates, and purified (Table 2). For mix-wine, all colonies (n°18) present in one countable plate seeded with the highest dilution (10^−4^) were isolated (Table 2). According to ITS-RFLP analysis, yeast cultures could be clustered in seven groups (Supplementary Table S1). Forty-nine isolates were identified as *S. cerevisiae* on the basis of their ITS-RFLP patterns (Supplementary Table S1). Non-*Saccharomyces* entities were all subjected to ITS sequence analysis to confirm presumptive identification obtained according to ITS-RFLP (Supplementary Table S1). Yeast species isolated in each sample are summarized in Table 2. Since all types of colonies were selected, even those showing slight differences on WL agar, a medium supposed to be highly differential (Pallmann et al., 2001); and since colonies were all picked by plates seeded with the highest dilutions, species recorded could be confidently considered as components of the dominant cultivable microbiota in that environment. Specifically, in must samples characterized by an alcoholic degree higher than 5% (musts 1, 8, 10, 11, and 12), only *S. cerevisiae* or *S. cerevisiae* and *H. uvarum* (must 8) were isolated. In other samples, *S. cerevisiae* was co-isolated with at least further two yeast species (musts 2, 3, 4, and 7) or was not detected (musts 5, 6, and 9). In the latter case, yeast microbiota of musts appeared to be characterized by a mix yeast population (*P. kudriavzevii, L. thermotolerans*, and *H. uvarum* or *M. pulcherrima* and *L. thermotolerans*) or by a single species (*M. pulcherrima*) (Table 2).

As expected, in mix wine, with an alcoholic degree of 12.8%, only isolates referable to *S. cerevisiae* species were retrieved. The 49 *S. cerevisiae* isolates (38 from must samples and 18 from mix wine) were typed by Interdelta analysis to evaluate their genetic diversities and to determine their clonal relationships. Supplementary Figure S1 shows patterns displayed by *S. cerevisiae* isolates detected in mix-wine at the end of fermentation. In must samples (n°38), a total of 13 different biotypes were detected (Table 3, patterns “I”–“XIII”). In several musts (1, 2, 8, 10, 11, and 12) more than one *S. cerevisiae* biotype occurred. Nevertheless, in some cases, the same biotype was detected in different samples, i.e., “V” in musts 3 and 8; “VII” in musts 7 and 8; “XII” in musts 11 and 12 (Table 3). Moreover, it is noteworthy that must samples 3, 7, 8, and 11, 12 were produced from grapes collected in closely located vineyards (Table 1). In mix wine, a total of eight different biotypes, out of 18 isolates, were retrieved: three (“IV,” “VII,” and “XII”) already detected in must samples and five new (“XIV”–“XVIII”) (Table 3). The biotype “XIV” showed the highest occurrence: 10 isolates out 18 analyzed.

**Table 3:** **Distribution of *S. cerevisiae* biotypes, detected by Interdelta analysis, in analyzed samples**.

**Must**	**No. of isolates**	***S. cerevisiae*** **biotypes**																	
		**I**	**II**	**III**	**IV**	**V**	**VI**	**VII**	**VIII**	**IX**	**X**	**XI**	**XII**	**XIII**	**XIV**	**XV**	**XVI**	**XVII**	**XVIII**
1	4	2^a^	2																
2	4			2	2														
3	2					2													
4	2						2												
7	2							2											
8	5					3		2											
10	4								1	3									
11	4										1	1	2						
12	4												3	1					
Mix-wine	18				1			1					1		10	2	1	1	1

a *Number of isolates showing the same Interdelta pattern*.

A total of 43 isolates (22 non-*Saccharomyces*, 13 *S. cerevisiae* isolates from musts samples and eight from mix-wine) were characterized for biochemical features of oenological interest (Table 4). Strains belonging to the same species showed similar ethanol resistance: *M. pulcherrima* (4–5%)*; Pseud. aphidis* (a yeast like fungi, classified in the *Ustilaginales*) (6%)*; H. uvarum* (7%); *Star. bacillaris* (synonym *Candida zemplinina*), and *L. thermotolerans* (10%); *P. kudriavzevii* (10–12%)*; S. cerevisiae* (15–16%). All strains were able to grow in YPD (pH 3.30) containing 200 mg/l of potassium metabisulfite and most of them grew in tyndallized must as dispersed cells. One isolate (T28) of *P. kudriavzevii* grew on the surface; while the two *Pseud. aphidis* cultures were flocculent (Table 4). *H. uvarum* strains were low H_2_S producers, *M. pulcherrima* fair producers, while the *Star. bacillaris* culture and those belonging to the species *Pseud. aphidis* were high producers (Table 4). Behavior within the species *P. kudriavzevii, L. thermotolerans*, and *S. cerevisiae* proved to be strain-dependent. Antagonistic activity was expressed by some isolates of *M. pulcherrima* (two out five)*, L. thermotolerans* (four out six), *P. kudriavzevii* (one out three), and *S. cerevisiae* (6 out 21). Cellobiose was hydrolyzed only by *M. pulcherrima* and *H. uvarum* isolates; while arbutin just by *M. pulcherrima*. For the other beta-glucosides used as precursors (4-methylumbelliferyl-b-D-glucopyranoside, esculin, and p-nitrophenyl β-D-glucopyranoside) different attitudes were recorded depending on the strain: the *Star. bacillaris* strain showed a low response, while *Pseud. aphidis* strains exhibited an high beta-glucosidase activity on these substrate (Table 4). Finally, only in *Pseud. aphidis* strains expressed esterase activity on a Tween 80-based medium.

**Table 4:** **Technological characteristics of yeasts collected during this study**.

**Species**	**No. of isolates**	**^a^Ethanol resistance**	**^b^K_2_O_5_S_2_ resistance**	**^c^Type of growth**	**^d^H_2_S production**	**^e^Antagonistic activity**	**Enzymatic activities**					
							**^f^CELL**	**^f^ARB**	**^f^ESC**	**^f^MUG**	**^f^pNPG**	**^g^EST**
*M. pulcherrima*	5	4, 5	200	D	M	2	+	+	M	L, M, H	M	−
*Star. bacillaris*	1	10	200	D	H	−	−	−	L	L	L	−
*P. kudriavzevii*	3	10-12	200	D, S	M, H	1	−	−	L, H	M, H	L, M	−
*L. thermotolerans*	6	10	200	D	M, H	4	−	−	L	L, M, H	L	−
*H. uvarum*	5	6, 7	200	D	L	-	+	−	M, H	H	L, M	−
*Pseud. aphidis*	2	6	200	F	H	-	−	−	H	H	H	+
*S. cerevisiae* (musts)	13	15, 16	200	D	M, H	5	−	−	M, H	M, H	L	−
*S. cerevisiae* (mix wines)	8	15, 16	200	D	M, H	1	−	−	M	M, H	L, M	−

a *In YPD broth ethanol-added (4–16% vol)*.

b *In YPD broth K_2_O_5_S_2_–added (50–200 mg/l—50 mg/l increments)*.

c *In tyndallized must (21°Brix, pH 3.50) after 4 days at 25°C: D, dispersed cells; S, surface growth; F, flocculent*.

d *On Biggy agar (Oxoid): L, low (Snow—White); M, medium (Hazelnut—Brown); H, high (Rust—Coffee)*.

e *Number of isolates/strains showing antagonistic activity (Sangorrin et al., 2001)*.

f *β-glucosidase activity evaluated on cellobiose (CELL) 4-methylumbelliferyl-b-D-glucopyranoside (MUG), arbutin (ARB), esculin (ESC) and p-Nitrophenyl β-D-glucopyranoside (pNPG) (Hernandez et al., 2002; Fia et al., 2005): +, positive; –, negative; L, low activity; M, medium activity; H, high activity*.

g *Esterase activity on Tween 80 (Slifkin, 2000)*.

**Figure 1:**
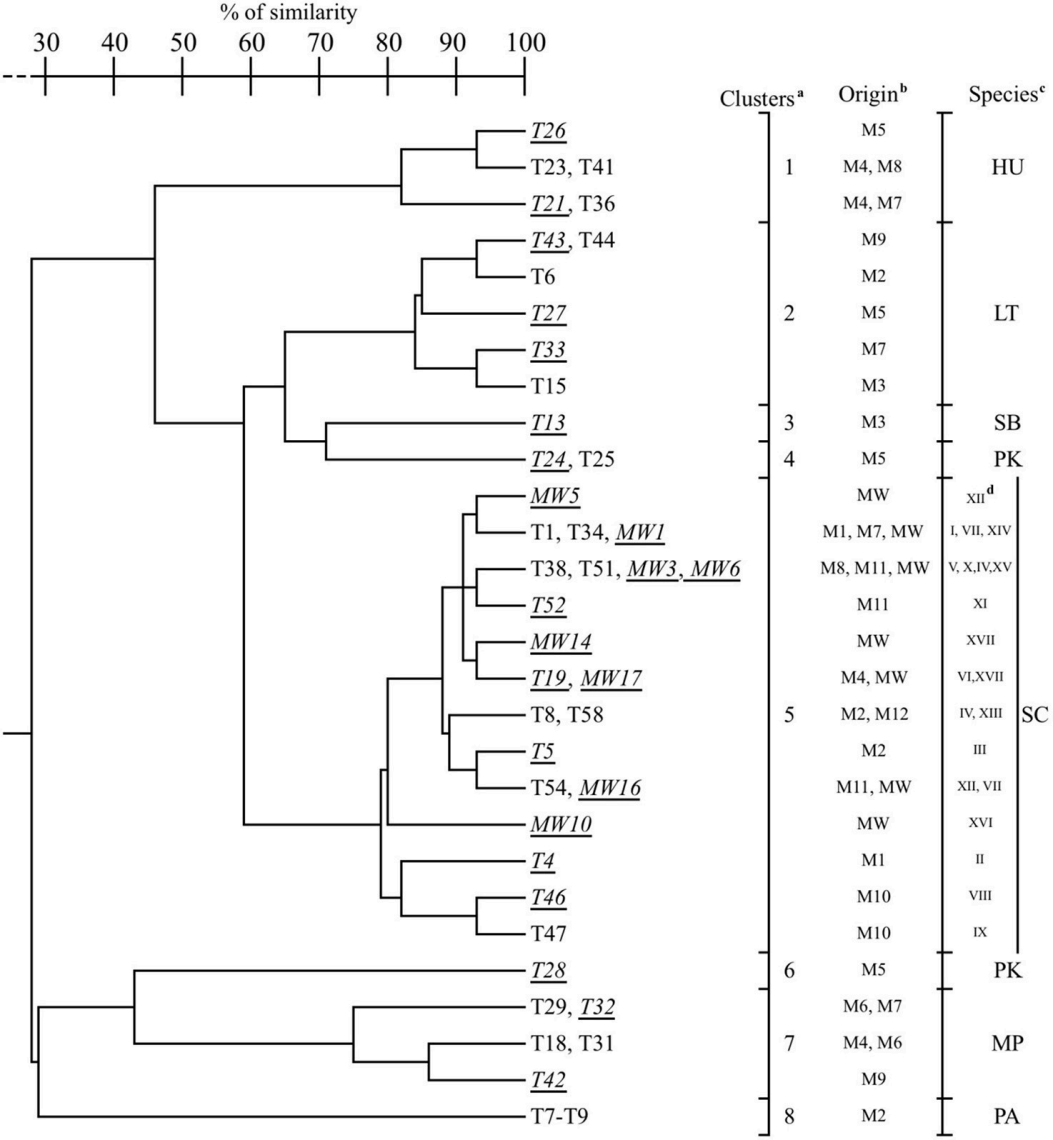
**UPGMA dendrogram obtained from the comparison of yeasts technological traits (see Table 4)**. ^a^Clusters were defined at 75% of similarity. ^b^Origin of isolates: M, must samples (see Table 1); MW, mix-wine. ^c^MP, *M. pulcherrima*; SB, *Star. bacillaris*; PK, *P. kudriavzevii*; LT, *L. thermotolerans*; HU, *H. uvarum*; PA, *Pseud. aphidis;* SC, *S. cerevisiae*. ^d^Interdelta biotypes of *S. cerevisiae* (I-XVIII). Isolates in underlined and *italicized* were used in the microfermentation trials (see Table 5).

The percentage of similarity among isolates, on the basis of technological traits, was evaluated by cluster analysis (Average Linkage Method) and the UPGMA dendrogram depicted in Figure 1 was obtained. Isolates of the same species clustered at a similarity level higher than 75%, with the unique exception of *P. kudriavzevii* strains which were positioned in two different clusters: T24 and T25 in cluster 4 and T28 in cluster 6. Actually, T28 differed from the other two isolates for ethanol resistance (12%), type of growth (superficial), H_2_S production (high), antagonistic activity (positive), and high beta-glucosidase activity (Table 4). In spite of the different origin and of the genetic diversity as emerged by Intedelta analysis, strains of *S. cerevisiae* grouped in a single cluster (cluster 5) with a quite high similarity level (80%) (Figure 1). No direct correlation between cluster position and origin of isolates was pointed out, even if, in some cases strains, strains with the same origin (T51 and T52, T8 and T5, T46 and T47; isolated from M11, M2, and M10, respectively) clustered very closely (>90 %) (Figure 1). Surprisingly, strains showing the same Interdelta pattern (T8 and MW3, pattern “IV”; T34 and MW16, pattern “VII”; T54 and MW5, pattern “XII”) showed technological traits poorly different (Figure 1). Combining data of Table 4 and Figure 1, 23 strains (10 non-*Saccharomyces* and 13 *S. cerevisiae*) were selected for the evaluation of the fermentation performances in Aglianico must containing about 240 g/l of reducing sugars and, therefore, an ethanolic potential of about 14% (vol/vol) (Table 5). Despite of their high beta-glucosidase and esterase activity, *Pseud. aphidis* strains were excluded because did not show fermentative activity. With exception of *P. kudriavzevii* isolates, strains of the same species showed similar FV values (*M. pulcherrima* 1.02–1.26 g CO_2_/100 ml; *H. uvarum* 2.12–2.13; *Star. bacillaris* 2.84; *L. thermotolerans* 3.88-4.01; *S. cerevisiae* 5.11–5.89) (Table 5). *M. pulcherrima* and *H. uvarum* strains showed a FP value lower of 4 g CO_2_/100 ml; all *L. thermotolerans* strains, the unique strain of *Star. bacillaris* and the strain T24 of *P. kudriavzevii* exhibited values ranging from 6.50 to 7.30; while *P. kudriavzevii* T28 a value of about 8.30. *S. cerevisae* strains, as expected, showed higher FP values, if compared to non-*Saccharomyces* (from 8.78 to 10.04 g CO_2_/100 ml). By HPLC analysis of wines at the end of fermentation (no weight change of fermentation flasks, in 48 h), *M. pulcherrima* and of *H. uvarum* strains were able to produce < 5% of ethanol (Table 5). However, both strains of *M. pulcherrima* produce undetectable (< 0.15 g/l) amounts of acetic acid, a very high quantity of succinic acid (about 10.5 g/L), and a medium level of glycerol (about 5.5 g/l). By contrast, *H. uvarum* strains produced 1.0–1.2 g/l of acetic acid, 1.0–1.2 g/l of succinic acid and a lower amount of glycerol (4.1–4.7 g/l). *Star. bacillaris* strain T13 was able to produce a wine with about 10% of ethanol (9.91 ± 0.24), and to entirely metabolize fructose and malic acid. By contrast, the wine still contained unfermented glucose (about 60 g/l), glycerol (9.3 g/l), and acetic acid (0.8 g/l). In spite of the similar ethanol content (10.7–11.0%) *P. kudriavzevii* strains produced different fermentation by-products. In fact, the wine produced by strain T28 still contained about 45 g/l of glucose, 1.1 g/l of acetic and succinic acids and about 7.6 g/l of glycerol; while, that produced by strain T24 contained even more unfermented sugars (about 86 g/l), glycerol (6.6 g/l), lactic (0.8 g/l), and succinic acid (3.1 g/l) and did not contain detectable amount of acetic acid. *L. thermotolerans* produced slightly different wines depending on the strain: ethanol content ranged from 9.5 to 10.5%, residual sugars from 60 to 70 g/l, succinic acid from 2.3 to 3.0 g/l, lactic acid from 1.3 to 2.5 g/l, while glycerol was always around 6.5 g/l and acetic acid remained undetectable (< 0.15 g/l). In wines produced by *S. cervisiae* strains some differences, depending on the strain used, emerged too. Two strains (MW16 and MW1), out 13, produced wines with a significant amount of unfermented fructose (15–20 g/l) and, as a consequence, with an alcoholic degree lower than 13% (11.92 and 12.75%, respectively). In the other, cases reducing sugars were detectable at low concentration (< 4 g/l) (wines produced by strains T52 and MW6) or undetectable (Table 5). In fact, the mean alcoholic degree of wines, excluding those produced by strains MW16 and MW1, was 13.8 ± 0.28% (minimum 13.19 ± 0.25 %, maximum 14.17 ± 0.19%). The acetic acid production by *S. cerevisiae* strains ranged from 0.52 (T19) to 1.86 g/l (MW10), even if, more than 50% of the strains produced < 0.6 g/l. Differences about glycerol production were also detected among wines produced by different strains of *S. cerevisiae*: from 5.35 of strain MW6 to 8.92 g/l of MW10, the high acetic acid producer. However, 60% of strains produced < 6 g/l of glycerol. Different amount of succinic acid were produced by *S. cerevisiae* strains: from 0.92 (MW5) to 2.25 g/l (MW16). Finally, no significant differences in tartaric acid content were observed among wines produced by the different strains; by contrast, malic acid content of wines produced by strains MW17 and MW3 was significantly different: 3.63 ± 0.22 and 5.49 ± 0.49 g/l, respectively.

**Table 5:** **Fermentation performances of selected yeast strains in Aglianico must**.

**Species**	**Strain**	**Weight loss**^**a**^		**HPLC analysis**^**b**^								
		**FV**	**FP**	**TART**	**GLU**	**FRU**	**MAL**	**SUC**	**LACT**	**GLYC**	**ACET**	**ETHA**
*M. pulcherrima*	T32	1.02	3.35	4.74 ± 0.27	45.8 ± 0.5	65.0 ± 0.5	NQ^c^	10.58 ± 0.36	<0.15	5.31 ± 0.05	<0.15	4.20 ± 0.07
*M. pulcherrima*	T42	1.26	3.70	4.79 ± 0.40	52.8 ± 0.5	89.0 ± 0.5	NQ	10.71 ± 0.28	<0.15	5.73 ± 0.15	<0.15	4.53 ± 0.03
*Star. bacillaris*	T13	2.84	6.97	4.00 ± 0.16	59.3 ± 0.5	<0.25	<0.25	0.83 ± 0.24	<0.15	9.34 ± 0.10	0.76 ± 0.01	9.91 ± 0.24
*P. kudriavzevii*	T28	2.17	8.25	4.40 ± 0.17	2.70 ± 0.23	43.9 ± 0.5	NQ	1.14 ± 0.15	<0.15	7.64 ± 0.21	1.15 ± 0.27	11.02 ± 0.35
*P. kudriavzevii*	T24	4.01	6.70	4.71 ± 0.21	30.6 ± 0.5	56.6 ± 0.5	NQ	3.14 ± 0.36	0.84 ± 0.30	6.66 ± 0.55	<0.15	10.74 ± 0.16
*L. thermotolerans*	T27	3.90	6.49	4.79 ± 0.33	28.1 ± 0.5	52.1 ± 0.5	NQ	2.28 ± 0.21	1.29 ± 0.43	6.45 ± 0.14	<0.15	10.42 ± 0.23
*L. thermotolerans*	T33	3.88	6.91	5.01 ± 0.21	23.1 ± 0.5	43.8 ± 0.5	NQ	3.03 ± 0.17	1.24 ± 0.52	6.33 ± 0.05	<0.15	9.46 ± 0.54
*L. thermotolerans*	T43	4.01	7.26	4.86 ± 0.55	19.2 ± 0.5	40.8 ± 0.5	NQ	2.55 ± 0.28	2.56 ± 0.65	6.71 ± 0.36	<0.15	10.24 ± 0.35
*H. uvarum*	T26	2.13	3.45	4.35 ± 0.22	70.0 ± 0.5	77.9 ± 0.5	NQ	1.01 ± 0.07	<0.15	4.77 ± 0.18	1.08 ± 0.03	4.41 ± 0.15
*H. uvarum*	T21	2.12	3.58	4.70 ± 0.47	69.2 ± 0.5	69.2 ± 0.5	NQ	1.28 ± 0.25	<0.15	4.12 ± 0.16	1.20 ± 0.16	4.36 ± 0.05
*S. cerevisiae*	T4	5.77	9.64	5.40 ± 0.59	<0.25	<0.25	4.03 ± 0.36	1.23 ± 0.03	<0.15	6.02 ± 0.21	0.55 ± 0.00	13.82 ± 0.14
*S. cerevisiae*	T5	5.83	9.53	5.60 ± 0.12	<0.25	<0.25	4.25 ± 0.42	1.67 ± 0.36	<0.15	5.61 ± 0.23	0.70 ± 0.06	13.89 ± 0.13
*S. cerevisiae*	T19	5.59	8.38	5.42 ± 0.13	<0.25	<0.25	4.15 ± 0.29	1.50 ± 0.03	<0.15	5.92 ± 0.15	0.52 ± 0.03	14.12 ± 0.26
*S. cerevisiae*	T46	5.49	9.50	5.79 ± 0.04	<0.25	<0.25	4.96 ± 0.31	2.08 ± 0.04	<0.15	6.19 ± 0.11	0.78 ± 0.01	13.78 ± 0.06
*S. cerevisiae*	T52	5.79	9.43	5.81 ± 0.00	1.44 ± 0.36	<0.25	4.20 ± 0.39	1.71 ± 0.21	<0.15	5.68 ± 0.19	0.77 ± 0.14	13.63 ± 0.21
*S. cerevisiae*	MW1	5.25	8.78	5.63 ± 0.48	1.21 ± 0.27	15.20 ± 0.42	4.75 ± 0.38	1.69 ± 0.26	<0.15	6.88 ± 0.09	0.95 ± 0.07	12.75 ± 0.12
*S. cerevisiae*	MW3	5.68	9.55	5.76 ± 0.46	<0.25	<0.25	5.49 ± 0.49	1.36 ± 0.35	<0.15	5.89 ± 0.01	0.53 ± 0.01	13.60 ± 0.38
*S. cerevisiae*	MW5	5.26	9.51	4.83 ± 0.14	<0.25	<0.25	4.38 ± 0.14	0.92 ± 0.62	<0.15	5.67 ± 0.12	0.61 ± 0.04	13.79 ± 0.08
*S. cerevisiae*	MW6	5.67	9.42	5.14 ± 0.51	<0.25	3.68 ± 0.01	4.60 ± 0.28	1.27 ± 0.02	<0.15	5.35 ± 0.17	0.68 ± 0.00	14.00 ± 0.29
*S. cerevisiae*	MW10	5.11	9.38	5.36 ± 0.32	<0.25	<0.25	4.59 ± 0.47	1.19 ± 0.29	<0.15	8.92 ± 0.16	1.86 ± 0.16	13.19 ± 0.25
*S. cerevisiae*	MW14	5.75	9.48	5.40 ± 0.26	<0.25	<0.25	4.05 ± 0.53	1.34 ± 0.13	<0.15	6.31 ± 0.00	0.61 ± 0.00	14.17 ± 0.19
*S. cerevisiae*	MW16	5.89	8.93	5.67 ± 0.00	<0.25	19.70 ± 0.66	4.62 ± 0.38	2.25 ± 0.03	<0.15	8.01 ± 0.34	0.57 ± 0.03	11.92 ± 0.25
*S. cerevisiae*	MW17	5.75	10.04	5.43 ± 0.06	<0.25	<0.25	3.63 ± 0.22	1.59 ± 0.34	<0.15	5.93 ± 0.25	0.56 ± 0.05	13.77 ± 0.14

*Results are reported as mean (±SD)*.

a *FV, fermentation vigor (g CO_2_/100 ml of must in 72 h). FP, Fermentation power (gr. CO_2_/100 ml of must until the end of fermentation)*.

b *TART, tartaric acid (g/l); GLU, glucose (g/l); FRU, fructose (g/l); MAL, malic acid (g/l); SUC, succinic acid (g/l); LACT, lactic acid (g/l); GLYC, glycerol (g/l); ACET, acetic acid (g/l); ETHA, ethanol (% vol/vol)*.

c *NQ, unquantifiable, due to the high residual fructose concentration*.

## Discussion

As recently reviewed by Barata et al. (2012), grapes are characterized by a complex microbial ecology including filamentous fungi, yeasts, and bacteria with different physiological characteristics that mightily affect wine quality. Some species (parasitic fungi and environmental bacteria) may be only found in grapes, while others microorganisms, such as yeast, lactic acid bacteria, and acetic acid bacteria, may survive and/or grow during winemaking process. The ratio occurring among groups of microorganisms depends on different ecology factors: climate conditions, viticulture practices, grape ripening stage, and health status of grapes that direct influences the availability of nutrients available for the epiphytic microflora. As matter of fact, health status is the main factor affecting the microbial ecology of grapes: damaged grapes possess higher microbial numbers and greater species diversity if compared to the healthy ones (Barata et al., 2012). This study focused on grape yeast microbiota able to survive and or to grow during both middle and final stages of wine fermentation, and, therefore, potentially able to impact on wine quality. Aglianico grape samples were collected in different vineyards full covering the production area of the Taurasi DOCG. Grapes may potentially host different genera of non-*Saccharomyces* yeasts mostly belonging to the following genera: *Metschnikowia, Dekkera, Pichia, Candida, Hanseniaspora, Kluyveromyces, Issatchenkia, Torulaspora, Debaryomyces, Saccharomycodes, Zygosaccharomyces*, and *Schizosaccharomyces* spp. (Mills et al., 2008). In must, strains of these genera are subjected to a selective pressure exerted by different factors including: high sugar content, high acidity, nutrient availability, low oxygen tension, increasing ethanol concentrations, and presence of specific inhibitors (SO_2_, botriticin, medium chain fatty acids) (Ribereau-Gayon et al., 2006; Mills et al., 2008). Therefore, after few days of fermentation, the occurrence of grape yeasts may vary depending on the must characteristics. Thereafter, in natural fermentation, is expected that *S. cerevisiae* (poorly occurring on grape) become dominant due to its high adaptation of must-wine environment. Despite of their progressive reduction during wine fermentation, non-*Saccharomyces* yeasts are considered important members of must-wine ecosystem and able to increase the “complexity” of the wines sensory profiles through the production of a massive range of sensory-active compounds, actually higher than that usually associated to *Saccharomyces* alone (Fleet, 2008). At technological maturity (°Babo higher then 19) Aglianico grapes still contain a high titratable acidity and low pH (Gambuti et al., 2009). Musts produced by grapes sampled during this study were characterized by different titratable acidity (8.6–11.2 g/l), pH (3.10–3.40), and sugar content (19.0–21.6). Moreover, due to their different origin of grapes samples, musts may likely contain different amounts of available nitrogen, phenolic compounds, pesticide residues, and fermentation inhibitors, also. Such diversity may be partly explained by the chemical and microbiological differences detected among the musts after 9 days of fermentation. The applied strategy allowed to detect both *Saccharomyces* and non-*Saccharomyces* entities. Species retrieved during this study, with the exception of *Pseud. aphids*, were frequently detected on grapes, cellar equipment and along wine fermentations (Mills et al., 2008). *Pseudozyma* spp., yeast-like fungi (*Ustilaginales*), mostly epiphytic or saprophytic, not pathogenic to plants (Buxdorf et al., 2013) and presumably disseminated by migratory birds (Francesca et al., 2012), have already been detected on grape berries (Pantelides et al., 2015), and just once, by a culture-independent approach PCR-DGGE based, in commercial wines (Takahashi et al., 2014). In the present study, members of this genus were retrieved in one out of 12 musts at early stage of fermentation. Even if strains detected during this study showed high esterase and beta-glucosidase activities, they do not seem to play any oenological role. However, *Pseudozyma* species have been reported to exhibit biological activity against powdery mildews and *Botrytis cinerea* (Buxdorf et al., 2013) and, due to their enzymatic activities, may represent an important source of microbial lipases, surfactants (Dimitrijević et al., 2011; Dziegielewska and Adamczak, 2013) and glucosidases (this study).

Because of their several negative fermentation characteristics, such as low fermentation power and rate, low SO_2_ resistance, and high production of acetic acid, ethyl acetate, acetaldehyde, and acetoin, non-*Saccharomyces* wine yeasts have been little considered as starter cultures in the past. However, as pointed out by this study, and previously by Comitini et al. (2011), some oenological traits of wine yeasts are species-specific (as ethanol resistance) and some are strain-specific (SO_2_ resistance, type of growth, killer factor expression, H_2_S production; enzymatic activities). Therefore, the strains selection among non-*Saccharomyces* may represent a profitable strategy to improve particular characteristics of wine (Suarez-Lepe and Morata, 2012). Despite of their low ethanol tolerance, as here reported, *M. pulcherrima* strains may exert antagonistic activity, high beta-glucosidase activity, low acetic acid production (Comitini et al., 2011), and high succinic acid accumulations (this study). *M. pulcherrima* strains may inhibit the growth of some spoilage yeasts (*Brettanomyces/Dekkera, Hanseniaspora*, and *Pichia*) (Oro et al., 2014) by pigment formation, which depletes the free iron in the medium thus generating an environment unsuitable for microorganisms requiring such element for the growth (Sipiczki, 2006).

Isolates of *H. uvarum* analyzed during this study proved to be high acetic acid producers, low H_2_S producers and potentially expressing beta-glucosidase activities. In fact, a recent study of Albertin et al. (2016) reports several extracellular enzymatic activities of oenological relevance (pectinase, chitinase, protease, β-glucosidase) in *H. uvarum* strains.

The two isolates of *P. kudriavzevii* (synonymously known as *Issatchenkia orientalis*) showed very different traits. Strain T28, showing antagonistic activity and showing the ability to hydrolyze esculine, MUG and pNPG, was able to produce a wine with 11% of ethanol, high concentration of acetic acid (1.1 g/l) and medium-high of glycerol (7.6 g/l). By contrast, strain T24 (antagonistic activity positive and beta-glucosidase negative) produced a wine with a similar alcoholic degree, containing undetectable amounts of acetic acid, low quantity of lactic acid and relatively high concentration of succinic acid. Killer toxin expression, lactic and succinic production were recently highlighted in strains of *P. kudriavzevii* (Bajaj et al., 2013; Xiao et al., 2014).

Strains of *L. thermotolerans* (formerly known as *Kluyveromyces thermotolerans*) produced wines with about 10% of ethanol, low acetic acid, high lactic, and relative high succinic acids, thus confirming data already reported by Comitini et al. (2011). Moreover, four out six strains were able to express killer toxin, while no strain analyzed by Comitini et al. (2011) expressed this character. Killer toxin production by *Kluyveromyces thermotolerans* IFO 1778 was reported by Kono and Himeno (1997).

This study also confirmed the fructophilic nature, the high glycerol production and the relative low ethanol and acetic acid synthesis by *Star. bacillaris* (synonym *Candida zemplinina*) during wine fermentation (Tofalo et al., 2012; Englezos et al., 2015). In addition, Tofalo et al. (2012) proved that strains of this species can also metabolized about 40% of the malic acid of must. *Star. bacillaris* strain T13, isolated during this study, was able to entirely metabolize malic acid; being this metabolite undetectable (< 0.25 g/l) by HPLC in the wine.

According to results, different biotypes of *S. cerevisiae* could be retrieved from the same grape sample; some biotypes could survive until the end of fermentation, while some other, not detectable in the grape or in must, become dominant in final product. In fact, as supposed by Sipiczki (2011), *S. cerevisiae* isolates of wine origin usually exhibit a significant biodiversity, due to the high propensity to genomic alteration of their genomes. In spite of the genetic diversity, *S. cerevisiae* strains exhibited an humble variability regarding their technological features and fermentation performances. Similar results were obtained by Capece et al. (2012): only three clusters out of 132 *S. cerevisiae* strains were obtained by statistical management of strains technological characterization. However, some strains isolated during this study showed undesirable characteristics as high H_2_S and acetic acid production, and high residual fructose in wine.

As recently reviewed by Capozzi et al. (2015), the utilization of non-*Saccharomyces*/*Saccharomyces* multi-starter has been suggested by different researchers in order to mimic the spontaneous fermentation process and to avoid the risks of stuck or sluggish fermentations; in fact, the last years numerous investigations dealt with the biodiversity of non-*Saccharomyces* yeast isolated from grape juice and their use in multi-starter fermentations. Moreover, there is an increasing demand for autochthonous yeast, with the aim to select starter cultures better adapted to a definite grape must, thus exploiting the biodiversity of a specific “*terroir*” (see Capozzi et al., 2015). As consequence, specific selection projects are required in order to prevent negative impact autochthonous yeast on wine fermentation and to exploit their beneficial contributions to wine quality. In this study, the yeasts diversity occurring in grapes of a restrict area where high quality wines are produced was explored. By evaluating oenological traits, the potential of some isolated strains (non-*Saccharomyces* and *S. cerevisae*) in combination to modulate quality of Taurasi DOCG wine was highlighted.

In conclusion, apart from the local relevance of the present study, obtained outcomes clearly confirm that *S. cerevisiae* is a member of the vineyard microbiota. Moreover, the hypothesis formulated by Sipiczki (2011) according to which the genome of *S. cerevisiae* can change during fermentation (Fast Adaptive Evolution) seems to gain a further proof.

Finally, results support the idea, already reported by several authors (Comitini et al., 2011; Rantsiou et al., 2012; Domizio et al., 2014; Zuehlke et al., 2015), that must fermentation with mixed cultures may improve the quality and complexity of the final product.

## Author contributions

GB designed the project. MA and GB performed all the experiments, wrote and edited the manuscript.

### Conflict of interest statement

The authors declare that the research was conducted in the absence of any commercial or financial relationships that could be construed as a potential conflict of interest.
