# Supplementary material for: Potential Role of Yeast Strains Isolated from Grapes in the Production of Taurasi DOCG
Source: Front Microbiol. 2016 May 27;7:809. doi: 10.3389/fmicb.2016.00809 (PMC4882326; doi:10.3389/fmicb.2016.00809)

**Supplementary tables**

**Table S1**. ITS, ITS-RFLP and ITS sequencing analyses of yeast isolates.

| **Isolate** | **Sample origin** | **ITS (bp)** | **ITS-RFLP size fragments (bp)** | | | | |  | **ITS sequencing data comparison with those of type strains*** | | | | |
| --- | --- | --- | --- | --- | --- | --- | --- | --- | --- | --- | --- | --- | --- |
|  |  |  | ***Hae* III** | ***Cfo* I** | ***Hinf* I** | ***Dde* I** | ***Mbo* I** |  | **Type strain** | | **Identities** | **Gaps** | **Identification** |
| T18 | 4 | 380 | 280/100 | 200/100/80 | 200/180 |  |  |  | CBS 5833^T^ | | 278/283 (98.2%) | 0 | *M. pulcherrima* |
| T29 | 6 | 380 | 280/100 | 200/100/80 | 200/180 |  |  |  |  |  | 278/283 (98.2%) | 0 | *M. pulcherrima* |
| T31 | 6 | 380 | 280/100 | 200/100/80 | 200/180 |  |  |  |  |  | 278/283 (98.2%) | 0 | *M. pulcherrima* |
| T32 | 7 | 380 | 280/100 | 200/100/80 | 200/180 |  |  |  |  |  | 279/283 (98.5%) | 0 | *M. pulcherrima* |
| T42 | 9 | 380 | 280/100 | 200/100/80 | 200/180 |  |  |  |  |  | 279/283 (98.5%) | 0 | *M. pulcherrima* |
| T13 | 3 | 480 | 480 | 215/110/100/60 | 250/230 |  | 300/150 |  | CBS 9494^T^ | | 405/409 (99.0%) | 0 | *C. zemplinina* |
| T24 | 5 | 500 | 360/100/40 | 210/150/70/50 | 200/160/140 |  |  |  | CBS 5174^T^ | | 267/269 (99.2 %) | 0 | *P. kudriavzevii* |
| T25 | 5 | 500 | 360/100/40 | 210/150/70/50 | 200/160/140 |  |  |  |  |  | 267/269 (99.2 %) | 0 | *P. kudriavzevii* |
| T28 | 5 | 500 | 360/100/40 | 210/150/70/50 | 200/160/140 |  |  |  |  |  | 267/269 (99.2 %) | 0 | *P. kudriavzevii* |
| T6 | 2 | 660 | 300/210/90/60 | 320/290/50 | 330/320 |  |  |  | CBS 6340^T^ | | 588/590 (99.6%) | 0 | *L. thermotolerans* |
| T15 | 3 | 660 | 300/210/90/60 | 320/290/50 | 330/320 |  |  |  |  |  | 588/590 (99.6%) | 1 | *L. thermotolerans* |
| T27 | 5 | 660 | 300/210/90/60 | 320/290/50 | 330/320 |  |  |  |  |  | 589/590 (99.7%) | 0 | *L. thermotolerans* |
| T33 | 7 | 660 | 300/210/90/60 | 320/290/50 | 330/320 |  |  |  |  |  | 588/590 (99.6%) | 0 | *L. thermotolerans* |
| T43 | 9 | 660 | 300/210/90/60 | 320/290/50 | 330/320 |  |  |  |  |  | 588/590 (99.6%) | 0 | *L. thermotolerans* |
| T44 | 9 | 660 | 300/210/90/60 | 320/290/50 | 330/320 |  |  |  |  |  | 590/590 (100 %) | 0 | *L. thermotolerans* |
| T21 | 4 | 750 | 750 | 320/300/110 | 330/190/160/60 | 290/160/90/70/50 |  |  | CBS 314^T^ | | 631/635 (99.3%) | 1 | *H. uvarum* |
| T23 | 4 | 750 | 750 | 320/300/110 | 330/190/160/60 | 290/160/90/70/50 |  |  |  |  | 631/635 (99.3%) | 1 | *H. uvarum* |
| T26** | 5 | 750 | 750 | 320/300/110 | 330/190/160/60 | 290/160/90/70/50 |  |  |  |  | 606/635 (95.4%) | 0 | *H.* aff. *uvarum* |
| T36 | 7 | 750 | 750 | 320/300/110 | 330/190/160/60 | 290/160/90/70/50 |  |  |  |  | 634/635 (99,8%) | 0 | *H. uvarum* |
| T41 | 8 | 750 | 750 | 320/300/110 | 330/190/160/60 | 290/160/90/70/50 |  |  |  |  | 633/635 (99,8%) | 1 | *H. uvarum* |
| T7 | 2 | 800 | 400/320/60 | 210/170/150/130/100 | 400/350 |  |  |  | CBS 517.83^T^ | | 626/630 (99.3%) | 2 | *Ps. aphidis* |
| T9 | 2 | 800 | 400/320/60 | 210/170/150/130/100 | 400/350 |  |  |  |  |  | 626/630 (99.3%) | 2 | *Ps. aphidis* |
| T4*** | various | 850 | 320/230/180/150 | 385/365/120 | 370/370/120 |  |  |  |  |  |  |  | *S. cerevisiae***** |

*ITS Sequences of type strains were retrieved from CBS data-base. **The ITS sequence of strain T26 showed only 94,4 % of identities (614/635) with that of *H. opuntiae* CBS 8733^T^. ***Plus further 48 isolates; ***Identification was achieved on the basis of ITS-RFLP analysis only.

**Supplementary figures**

**Figure S1:** Interdelta patterns showed by *S. cerevisiae* isolates retrieved from mix-wine. M: Molecular weight standard, 1 Kb plus Ladder (Invitrogen).

**Supplementary Figure S1**


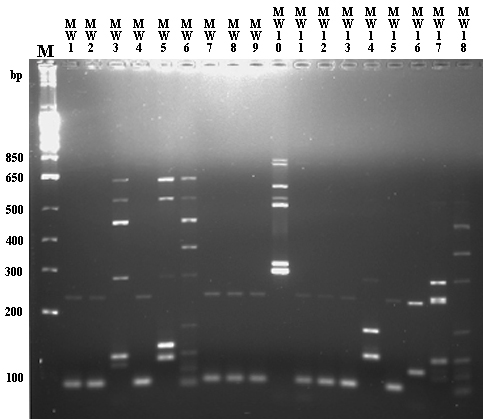

Supplement: Supplementary file 1 [file DataSheet1.doc]
